# Supplementary material for: Insights Into Symmetry and Substitution Patterns Governing Singlet–Triplet Energy Gap in the Chemical Space of Azaphenalenes
Source: Chemistry. 2026 Feb 5;32(15):e03557. doi: 10.1002/chem.202503557 (PMC13107512; doi:10.1002/chem.202503557)
Supplement: Supplementary file 1 — Supporting Information: (i) Geometries and additional data are available in the AP117 dataset [86], (ii) Table S1 presents names and SMILES representations, and (iii) Table S2 presents excitation energies and singlet–triplet gaps. [file CHEM-32-e03557-s001.pdf]

Supplementary information for:

Insights into Symmetry and Substitution Patterns Governing Singlet–Triplet Energy Gap in the Chemical Space of Azaphenalenes

Atreyee Majumdar and Raghunathan Ramakrishnan*

Tata Institute of Fundamental Research Hyderabad, Hyderabad 500046, India.

E-mail: ramakrishnan@tifrh.res.in

Table of Contents

1. **Dataset** Energies of the S_1 and T_1 states and the corresponding singlet–triplet gaps for all azaphenalenes investigated in this work, together with the optimized geometries obtained using the extrapolation scheme.

2. **Table S1** Names and SMILES representations of the azaphenalenes investigated in this work.

3. **Table S2** S_1 and T_1 excitation energies and singlet–triplet gaps calculated at the L-CC2/aug-cc-pVDZ level for all 104 azaphenalenes, including additional entries for systems exhibiting symmetry-lowering distortions.

Dataset

The AP117 dataset[1] provides optimized geometries for all azaphenalenes considered in this study, obtained using the extrapolation procedure described in Eq. (10) of the main text. For systems prone to symmetry lowering, both the high- and low-symmetry geometries are included. In addition, excitation energies of the S_1 and T_1 states and the corresponding singlet–triplet gaps, calculated using L-CC2 with the aug-cc-pVDZ basis set, are provided.

Table S1: Names and SMILES representations of the 104 azaphenalenes investigated in this work.

S.No.	Smiles	Name
1	C1=CC2=CC=CC3=CC=CC(=C1)N23	cyclazine
2	C1=CC2=CC=NC3=CC=CC(=C1)N23	1-aza
3	C1=CC2=CN=CC3=CC=CC(=C1)N23	2-aza
4	C1=CC2=CN=NC3=CC=CC(=C1)N23	1,2-biaza
5	C1=CC2=NC=NC3=CC=CC(=C1)N23	1,3-biaza
6	C1=NC2=CC=NC3=CC=CC(=C1)N23	1,4-biaza
7	N1=CC2=CC=NC3=CC=CC(=C1)N23	1,5-biaza
8	C1=CC2=CC=NC3=CC=CC(=N1)N23	1,6-biaza
9	C1=CC2=CC=NC3=CN=CC(=C1)N23	1,8-biaza
10	C1=CC2=CC=NC3=NC=CC(=C1)N23	1,9-biaza
11	N1=CC2=CN=CC3=CC=CC(=C1)N23	2,5-biaza
12	C1=CC2=NN=NC3=CC=CC(=C1)N23	1,2,3-triaza
13	C1=NC2=CN=NC3=CC=CC(=C1)N23	1,2,4-triaza
14	N1=CC2=CN=NC3=CC=CC(=C1)N23	1,2,5-triaza
15	C1=CC2=CN=NC3=CC=CC(=N1)N23	1,2,6-triaza
16	C1=CC2=CN=NC3=CC=NC(=C1)N23	1,2,7-triaza
17	C1=CC2=CN=NC3=CN=CC(=C1)N23	1,2,8-triaza
18	C1=CC2=CN=NC3=NC=CC(=C1)N23	1,2,9-triaza
19	C1=NC2=NC=NC3=CC=CC(=C1)N23	1,3,4-triaza
20	C1=CC2=NC=NC3=CC=NC(=C1)N23	1,3,6-triaza
21	C1=CC2=NC=NC3=CN=CC(=C1)N23	1,3,5-triaza
22	C1=NC2=CC=NC3=CC=NC(=C1)N23	1,4,7-triaza
23	C1=NC2=CC=NC3=CN=CC(=C1)N23	1,4,8-triaza
24	C1=NC2=CC=NC3=NC=CC(=C1)N23	1,4,9-triaza
25	N1=CC2=CC=NC3=CN=CC(=C1)N23	1,5,8-triaza
26	N1=CC2=CC=NC3=NC=CC(=C1)N23	1,5,9-triaza
27	C1=CC2=CC=NC3=CN=CC(=N1)N23	1,6,8-triaza
28	N1=CC2=CN=CC3=CN=CC(=C1)N23	2,5,8-triaza
29	C1=NC2=NN=NC3=CC=CC(=C1)N23	1,2,3,4-tetraaza
30	N1=CC2=NN=NC3=CC=CC(=C1)N23	1,2,3,5-tetraaza
31	C1=CC2=NN=NC3=CC=CC(=N1)N23	1,2,3,6-tetraaza
32	N1=NC2=CN=NC3=CC=CC(=C1)N23	1,2,4,5-tetraaza
33	C1=NC2=CN=NC3=CC=CC(=N1)N23	1,2,4,6-tetraaza
34	C1=NC2=CN=NC3=CC=NC(=C1)N23	1,2,4,7-tetraaza
35	C1=NC2=CN=NC3=CN=CC(=C1)N23	1,2,4,8-tetraaza
36	C1=NC2=CN=NC3=NC=CC(=C1)N23	1,2,4,9-tetraaza
37	N1=CC2=CN=NC3=CC=CC(=N1)N23	1,2,5,6-tetraaza
38	N1=CC2=CN=NC3=CC=NC(=C1)N23	1,2,5,7-tetraaza
39	N1=CC2=CN=NC3=CN=CC(=C1)N23	1,2,5,8-tetraaza
40	N1=CC2=CN=NC3=NC=CC(=C1)N23	1,2,5,9-tetraaza
41	C1=CC2=CN=NC3=CC=NC(=N1)N23	1,2,6,7-tetraaza

Continued from previous page		
S.No.	Smiles	Name
42	C1=CC2=CN=NC3=CN=CC(=N1)N23	1,2,6,8-tetraaza
43	C1=CC2=CN=NC3=NC=CC(=N1)N23	1,2,6,9-tetraaza
44	C1=CC2=CN=NC3=NC=NC(=C1)N23	1,2,7,9-tetraaza
45	C1=CC2=CN=NC3=NN=CC(=C1)N23	1,2,8,9-tetraaza
46	C1=NC2=NC=NC3=CC=CC(=N1)N23	1,3,4,6-tetraaza
47	C1=NC2=NC=NC3=CC=NC(=C1)N23	1,3,4,7-tetraaza
48	C1=NC2=NC=NC3=CN=CC(=C1)N23	1,3,4,8-tetraaza
49	C1=NC2=NC=NC3=NC=CC(=C1)N23	1,3,4,9-tetraaza
50	N1=CC2=NC=NC3=CC=NC(=C1)N23	1,3,5,7-tetraaza
51	N1=CC2=NC=NC3=CN=CC(=C1)N23	1,3,5,8-tetraaza
52	C1=CC2=NC=NC3=CC=NC(=N1)N23	1,3,6,7-tetraaza
53	N1=NC2=NN=NC3=CC=CC(=C1)N23	1,2,3,4,5-pentaaza
54	C1=NC2=NN=NC3=CC=CC(=N1)N23	1,2,3,4,6-pentaaza
55	C1=NC2=NN=NC3=CC=NC(=C1)N23	1,2,3,4,7-pentaaza
56	C1=NC2=NN=NC3=CN=CC(=C1)N23	1,2,3,4,8-pentaaza
57	C1=NC2=NN=NC3=NC=CC(=C1)N23	1,2,3,4,9-pentaaza
58	N1=CC2=NN=NC3=CC=CC(=N1)N23	1,2,3,5,6-pentaaza
59	N1=CC2=NN=NC3=CC=NC(=C1)N23	1,2,3,5,7-pentaaza
60	N1=CC2=NN=NC3=CN=CC(=C1)N23	1,2,3,5,8-pentaaza
61	C1=CC2=NN=NC3=CC=NC(=N1)N23	1,2,3,6,7-pentaaza
62	N1=NC2=CN=NC3=CC=NC(=C1)N23	1,2,4,5,7-pentaaza
63	N1=NC2=CN=NC3=CN=CC(=C1)N23	1,2,4,5,8-pentaaza
64	N1=NC2=CN=NC3=NC=CC(=C1)N23	1,2,4,5,9-pentaaza
65	C1=NC2=CN=NC3=CC=NC(=N1)N23	1,2,4,6,7-pentaaza
66	C1=NC2=CN=NC3=CN=CC(=N1)N23	1,2,4,6,8-pentaaza
67	C1=NC2=CN=NC3=NC=CC(=N1)N23	1,2,4,6,9-pentaaza
68	C1=NC2=CN=NC3=NC=NC(=C1)N23	1,2,4,7,9-pentaaza
69	C1=NC2=CN=NC3=NN=CC(=C1)N23	1,2,4,8,9-pentaaza
70	N1=CC2=CN=NC3=CN=CC(=N1)N23	1,2,5,6,8-pentaaza
71	N1=CC2=CN=NC3=NC=CC(=N1)N23	1,2,5,6,9-pentaaza
72	N1=CC2=CN=NC3=NC=NC(=C1)N23	1,2,5,7,9-pentaaza
73	N1=CC2=CN=NC3=NN=CC(=C1)N23	1,2,5,8,9-pentaaza
74	C1=CC2=CN=NC3=NC=NC(=N1)N23	1,2,6,7,9-pentaaza
75	C1=NC2=NC=NC3=CC=NC(=N1)N23	1,3,4,6,7-pentaaza
76	C1=NC2=NC=NC3=CN=CC(=N1)N23	1,3,4,6,8-pentaaza
77	N1=NC2=NN=NC3=CC=CC(=N1)N23	1,2,3,4,5,6-hexaaza
78	N1=NC2=NN=NC3=CC=NC(=C1)N23	1,2,3,4,5,7-hexaaza
79	N1=NC2=NN=NC3=CN=CC(=C1)N23	1,2,3,4,5,8-hexaaza
80	N1=NC2=NN=NC3=NC=CC(=C1)N23	1,2,3,4,5,9-hexaaza
81	C1=NC2=NN=NC3=CC=NC(=N1)N23	1,2,3,4,6,7-hexaaza
82	C1=NC2=NN=NC3=CN=CC(=N1)N23	1,2,3,4,6,8-hexaaza
83	C1=NC2=NN=NC3=NC=CC(=N1)N23	1,2,3,4,6,9-hexaaza
84	C1=NC2=NN=NC3=CN=NC(=C1)N23	1,2,3,4,7,8-hexaaza

Continued from previous page

S.No.	Smiles	Name
85	N1=CC2=NN=NC3=CC=NC(=N1)N23	1,2,3,5,6,7-hexaaza
86	N1=CC2=NN=NC3=CN=CC(=N1)N23	1,2,3,5,6,8-hexaaza
87	N1=NC2=CN=NC3=CN=NC(=C1)N23	1,2,4,5,7,8-hexaaza
88	N1=NC2=CN=NC3=NC=NC(=C1)N23	1,2,4,5,7,9-hexaaza
89	N1=NC2=CN=NC3=NN=CC(=C1)N23	1,2,4,5,8,9-hexaaza
90	C1=NC2=CN=NC3=NC=NC(=N1)N23	1,2,4,6,7,9-hexaaza
91	C1=NC2=CN=NC3=NN=CC(=N1)N23	1,2,4,6,8,9-hexaaza
92	N1=CC2=CN=NC3=NC=NC(=N1)N23	1,2,5,6,7,9-hexaaza
93	C1=NC2=NC=NC3=NC=NC(=N1)N23	1,3,4,6,7,9-hexaaza
94	N1=NC2=NN=NC3=CC=NC(=N1)N23	1,2,3,4,5,6,7-heptaaza
95	N1=NC2=NN=NC3=CN=CC(=N1)N23	1,2,3,4,5,6,8-heptaaza
96	N1=NC2=NN=NC3=CN=NC(=C1)N23	1,2,3,4,5,7,8-heptaaza
97	N1=NC2=NN=NC3=NC=NC(=C1)N23	1,2,3,4,5,7,9-heptaaza
98	N1=NC2=NN=NC3=NN=CC(=C1)N23	1,2,3,4,5,8,9-heptaaza
99	C1=NC2=NN=NC3=CN=NC(=N1)N23	1,2,3,4,6,7,8-heptaaza
100	C1=NC2=NN=NC3=NC=NC(=N1)N23	1,2,3,4,6,7,9-heptaaza
101	N1=CC2=NN=NC3=CN=NC(=N1)N23	1,2,3,5,6,7,8-heptaaza
102	N1=NC2=NN=NC3=CN=NC(=N1)N23	1,2,3,4,5,6,7,8-octaaza
103	N1=NC2=NN=NC3=NC=NC(=N1)N23	1,2,3,4,5,6,7,9-octaaza
104	N1=NC2=NN=NC3=NN=NC(=N1)N23	1,2,3,4,5,6,7,8,9-nonaaza

Table S2: S_1 and T_1 excitation energies and singlet–triplet gaps for all 104 azaphenalenes calculated at the L-CC2/aug-cc-pVDZ level. For the 13 azaphenalenes that undergo symmetry-lowering distortions, values are reported for both the high- and low-symmetry geometries, resulting in a total of 117 entries.

S.No.	Name	Point Group	S_1	T_1	STG
1	1,3,4,6,7,9-hexaaza	D_{3h}	2.723	2.952	−0.229
2	1,2,3,4,5,6,7,8,9-nonaaza	D_{3h}	1.988	2.176	−0.188
3	1,3,6,7-tetraaza	C_{2v}	2.127	2.304	−0.177
4	1,3,4,6,7-pentaaza	C_s	2.466	2.632	−0.166
5	1,6-biaza	C_{2v}	1.560	1.701	−0.141
6	1,3,4,6-tetraaza	C_{2v}	2.190	2.326	−0.136
7	1,2,3,4,9-pentaaza	C_{2v}	1.914	2.049	−0.135
8	-Cyclazine	D_{3h}	1.033	1.167	−0.134
9	1,2,4,6,8,9-hexaaza	C_{2v}	1.779	1.901	−0.122
10	1,3,4,9-tetraaza	C_{2v}	2.126	2.245	−0.119
11	1,6,8-triaza	C_{2v}	1.434	1.551	−0.117
12	1,3-biaza	C_{2v}	1.643	1.758	−0.115
13	1,9-biaza	C_{2v}	1.583	1.696	−0.113
14	1,2,3,4,5,6-hexaaza	C_{2v}	1.722	1.833	−0.111
15	1,3,7-triaza	C_s	1.968	2.073	−0.105
16	1,2,3,5,6,7,8-heptaaza	C_{2v}	1.500	1.599	−0.099
17	1,2,3-triaza	C_{2v}	1.412	1.510	−0.098
18	1,2,3,4,5,6,8-heptaaza	C_{2v}	1.583	1.680	−0.097
19	1,2,3,6,7-pentaaza	C_{2v}	1.831	1.926	−0.095
20	1,4,9-triaza	C_s	1.924	2.015	−0.091
21	1,2,8,9-tetraaza	C_{2v}	1.255	1.344	−0.089
22	2-aza	C_{2v}	0.889	0.978	−0.089
23	1,2,4,6,9-pentaaza	C_s	2.016	2.095	−0.079
24	1,2,3,4,6,7-hexaaza	C_s	2.200	2.278	−0.078
25	1,2,3,4,6,9-hexaaza	C_s	2.323	2.400	−0.077
26	2,5-biaza	C_{2v}	0.737	0.808	−0.071
27	1,2,3,5,8-pentaaza	C_{2v}	1.141	1.207	−0.066
28	1,3,4,7-tetraaza	C_s	2.338	2.403	−0.065
29	1,2,3,4,6-pentaaza	C_s	1.979	2.042	−0.063
30	1,3,4-triaza	C_s	1.986	2.049	−0.063
31	2,5,8-triaza	D_{3h}	0.593	0.651	−0.058
32	1,2,6,9-tetraaza	C_s	1.655	1.709	−0.054
33	1,2,5,6,8-pentaaza	C_{2v}	1.043	1.090	−0.047
34	-Cyclazine	C_{3h}	1.181	1.228	−0.047
35	1,3,4,6,8-pentaaza	C_{2v}	2.045	2.091	−0.046
36	1,2,3,4,5,8,9-heptaaza	C_{2v}	1.558	1.603	−0.045
37	1,5,9-triaza	C_{2v}	1.415	1.444	−0.029
38	1,3,8-triaza	C_s	1.560	1.587	−0.027
39	1,2,3,4-tetraaza	C_s	1.825	1.851	−0.026

**Continued from previous page**					
S.No.	Name	Point Group	S_1	T_1	STG
40	1,3,5,8-tetraaza	C_{2v}	1.359	1.382	−0.023
41	1,2,5,8,9-pentaaza	C_{2v}	1.091	1.113	−0.022
42	1,2,5,6-tetraaza	C_{2v}	1.153	1.163	−0.01
43	1,3,5,7-tetraaza	C_s	1.932	1.940	−0.008
44	1,2,3,4,6,8-hexaaza	C_s	1.931	1.928	0.003
45	1,2,8,9-tetraaza	C_{2v}	1.448	1.439	0.009
46	1,2,3,4,5,6,7-heptaaza	C_s	2.241	2.226	0.015
47	1,2,4,6,7,9-hexaaza	C_s	2.463	2.445	0.018
48	1-aza	C_s	1.585	1.565	0.02
49	1,3,4,8-tetraaza	C_s	1.874	1.845	0.029
50	1,2,4,6-tetraaza	C_s	1.957	1.927	0.03
51	1,2,3,6-tetraaza	C_s	1.876	1.844	0.032
52	1,2,3,4,7-pentaaza	C_s	2.227	2.194	0.033
53	1,2,6,7,9-pentaaza	C_s	2.077	2.041	0.036
54	1,2,5,6,7,9-hexaaza	C_{2v}	1.727	1.683	0.044
55	1,2,3,4,5,6,8-heptaaza	C_{2v}	1.901	1.851	0.05
56	1,2,3,5,6,7-hexaaza	C_s	1.958	1.906	0.052
57	1,2,3,4,5,7,9-heptaaza	C_s	2.488	2.434	0.054
58	1,4-biaza	C_s	1.947	1.892	0.055
59	1,2,6-triaza	C_s	1.592	1.536	0.056
60	1,2,4,9-tetraaza	C_s	2.030	1.973	0.057
61	1,2,3,4,8-pentaaza	C_s	1.807	1.748	0.059
62	1,2,3,4,5,9-hexaaza	C_s	2.075	2.014	0.061
63	1,2,3,5-tetraaza	C_s	1.540	1.473	0.067
64	1,2,9-triaza	C_s	1.690	1.623	0.067
65	1,2,4,8,9-pentaaza	C_s	1.861	1.790	0.071
66	1,4,7-triaza	C_{3h}	2.310	2.236	0.074
67	1,2,3,5,6,7,8-heptaaza	C_{2v}	1.884	1.801	0.083
68	1,2,4,6,7-pentaaza	C_s	2.350	2.260	0.09
69	1,2,4,7,9-pentaaza	C_s	2.454	2.361	0.093
70	2-aza	C_s	1.224	1.129	0.095
71	1,2,3,4,6,7,9-heptaaza	C_{2v}	2.444	2.345	0.099
72	1,8-biaza	C_s	1.555	1.454	0.101
73	1,2,6,8-tetraaza	C_s	1.633	1.530	0.103
74	1,2,4,6,8-pentaaza	C_s	2.010	1.905	0.105
75	1,2,3,4,5,6,7,9-octaaza	C_{2v}	2.204	2.094	0.11
76	1,2,3,5,7-pentaaza	C_s	1.925	1.813	0.112
77	1,2,3,4,5,8,9-heptaaza	C_{2v}	1.899	1.783	0.116
78	1,2,5,6-tetraaza	C_{2v}	1.411	1.285	0.126
79	1,2,6,7-tetraaza	C_s	1.998	1.867	0.131
80	1,2,7,9-tetraaza	C_s	2.135	1.999	0.136
81	1,4,8-triaza	C_s	1.921	1.778	0.143
82	1,2,3,5,8-pentaaza	C_{2v}	1.582	1.435	0.147

Continued from previous page					
S.No.	Name	Point Group	S_1	T_1	STG
83	1,2,3,4,6,7,8-heptaaza	C_s	2.357	2.203	0.154
84	1,5-biaza	C_s	1.605	1.451	0.154
85	1,2,3,4,5-pentaaza	C_s	2.092	1.937	0.155
86	1,2,3,4,5,7-hexaaza	C_s	2.452	2.296	0.156
87	1,2,3,5,6-pentaaza	C_s	1.989	1.827	0.162
88	1,2,3,4,7,8-hexaaza	C_s	2.354	2.188	0.166
89	1,2,4-triaza	C_s	2.032	1.856	0.176
90	1,2,5,6,9-pentaaza	C_s	1.828	1.641	0.187
91	1,2,5,6,8-pentaaza	C_{2v}	1.511	1.323	0.188
92	1,2,5,8,9-pentaaza	C_{2v}	1.554	1.366	0.188
93	1,2,5,9-tetraaza	C_s	1.704	1.505	0.199
94	1,2,4,5,7,9-hexaaza	C_s	2.558	2.351	0.207
95	1,2,4,7-tetraaza	C_s	2.401	2.194	0.207
96	1,2,5,7,9-pentaaza	C_s	2.101	1.888	0.213
97	1,2-biaza	C_s	1.733	1.517	0.216
98	1,5,8-triaza	C_s	1.617	1.394	0.223
99	2,5-biaza	C_s	1.289	1.065	0.224
100	1,2,4,5,9-pentaaza	C_s	2.195	1.950	0.245
101	1,2,7-triaza	C_s	2.101	1.856	0.245
102	1,2,3,5,6,8-hexaaza	C_s	2.109	1.857	0.252
103	1,2,4,8-tetraaza	C_s	2.065	1.812	0.253
104	1,2,3,4,5,8-hexaaza	C_s	2.138	1.883	0.255
105	1,2,8-triaza	C_s	1.761	1.485	0.276
106	1,2,3,4,5,7,8-heptaaza	C_s	2.644	2.363	0.281
107	1,2,3,4,5,6,7,8,9-nonaaza	C_{3h}	2.425	2.129	0.296
108	1,2,4,5,8,9-hexaaza	C_s	2.093	1.787	0.306
109	1,2,4,5,7-pentaaza	C_s	2.572	2.250	0.322
110	1,2,5,7-tetraaza	C_s	2.123	1.801	0.322
111	1,2,3,4,5,6,7,8-octaaza	C_s	2.531	2.204	0.327
112	1,2,5-triaza	C_s	1.794	1.466	0.328
113	2,5,8-triaza	C_{3h}	1.372	1.035	0.337
114	1,2,4,5-tetraaza	C_s	2.250	1.902	0.348
115	1,2,5,8-tetraaza	C_s	1.833	1.448	0.385
116	1,2,4,5,8-pentaaza	C_s	2.309	1.890	0.419
117	1,2,4,5,7,8-hexaaza	C_{3h}	2.797	2.376	0.421

References

(1) Majumdar, A.; Ramakrishnan, R. AP117 [Data set]. 2025; https://doi.org/10.5281/zenodo.17567791.
